# Ganglioside lipids inhibit the aggregation of the Alzheimer's amyloid-β peptide†

**DOI:** 10.1039/d4cb00189c

**Published:** 2025-03-13

**Authors:** Zenon Toprakcioglu, Akhila K. Jayaram, Tuomas P. J. Knowles

**Affiliations:** a Yusuf Hamied Department of Chemistry, University of Cambridge Lensfield Road Cambridge CB2 1EW UK tpjk2@cam.ac.uk; b Cavendish Laboratory, Department of Physics, University of Cambridge J J Thomson Avenue Cambridge CB3 0HE UK

## Abstract

The aggregation of the amyloid-β (Aβ) peptides (Aβ42/Aβ40) into amyloid fibrils and plaques is one of the molecular hallmarks in dementia and Alzheimer's disease (AD). While the molecular mechanisms behind this aggregation process are not fully known, it has been shown that some biomolecules can accelerate this process whereas others can inhibit amyloid formation. Lipids, which are ubiquitously found in cell membranes, play a pivotal role in protein aggregation. Here, we investigate how ganglioside lipids, which are abundant in the brain and in neurons, can influence the aggregation kinetics of both Aβ42 and Aβ40. We employ a variety of biophysical assays to characterise the effect ganglioside lipids have on the aggregation of Aβ. Through kinetic analysis, we show that the primary nucleation rate is greatly affected by the addition of gangliosides and that these lipids impair Aβ42 aggregation, while completely inhibiting Aβ40 aggregation. Furthermore, we find that an Aβ-ganglioside complex is formed, which potentially disrupts the aggregation pathway and results in delayed kinetics. Taken together, our results provide a quantitative description of how lipid molecules such as gangliosides can inhibit the aggregation of Aβ and shed light on the key factors that control these processes. In view of the fact that declining levels of gangliosides in neurons have been associated with ageing, our findings could be instrumental towards establishing new approaches in the prevention of amyloid-β aggregation.

## Abstract 2

The aggregation of amyloid-β (Aβ) into fibrils/plaques is associated with Alzheimer's Disease. Lipids play a crucial role in this process. We show that ganglioside lipids inhibit Aβ40/Aβ42 aggregation. This could be used to prevent fibril formation.

## Graphical abstract

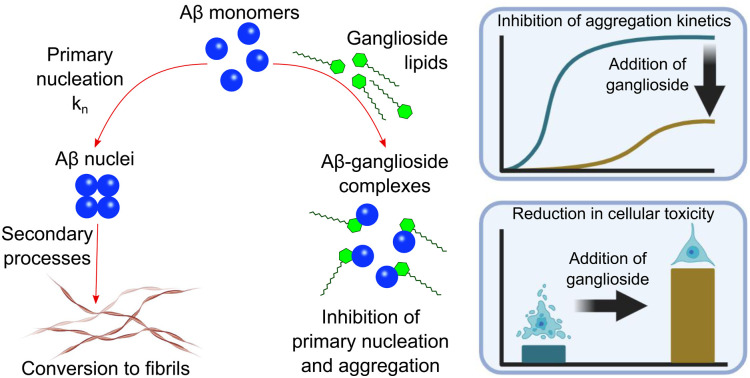

## Introduction

Alzheimers disease (AD) is the most common and prevalent neurodegenerative disorder accounting for 60–70% of all dementia cases.^1–3^ AD has been associated with the self-assembly and misfolding of physiologically generated proteins such as tau and amyloid-β (Aβ).^4–8^ The two major fragments of the Aβ peptide, Aβ42 and Aβ40, have been known to aggregate into fibrils, and ultimately amyloid plaques – a process which is regarded as the hallmark for AD.^7–9^ Furthermore, the aggregation of Aβ into such structures results in neuronal dysfunction and ultimately cell death, which subsequently leads to the pathology and progression of AD.^10,11^ Since the Aβ peptide is naturally found extracellularly, due to being cleaved-off from the membrane-bound amyloid precursor protein (APP),^2,11^ understanding the interaction of Aβ with lipid membranes and surfaces from a mechanistic viewpoint is extremely important. While it is known that different lipids can affect protein aggregation,^12,13^ either by inhibiting or accelerating the overall process, there is a lack of insight into the physicochemical mechanisms that ultimately determine which of these pathways will prevail.

A particular type of lipid which is abundant in the brain and in neuronal cells are gangliosides. These glycosphingolipids are essential in cell-to-cell communication, cell signaling and also play an important role in immunomodulation. Additionally, they are thought to be involved with neuroprotection, have neurotrophic properties^14,15^ and exhibit neurorestorative effects.^16,17^ Following their synthesis, gangliosides are transported to the outer leaflet where they form part of the plasma membrane and are almost exclusively found there.^18,19^ Their large hydrophilic head group protrudes into the extracellular environment, where they can interact with other biomolecules (proteins and/or lipids).^20^ Gangliosides are thus physiologically expressed in the outer layer of the membrane surface and given that Aβ is also physiologically present in the extracellular space, the interaction between Aβ and ganglioside lipids is especially relevant to study and understand. Although gangliosides are abundant in neurons, with concentrations reaching ten times higher than those found in non-neuronal cells, ganglioside levels progressively decrease with age.^21–24^ This could therefore be a contributing factor to why elderly people, who naturally possess lower ganglioside levels, are more prone to neurodegeneration and dementia. Numerous studies involving gangliosides and α-synuclein, a protein implicated and involved with Parkinson's disease (PD) and other synucleinopathies, have been conducted.^24–27^

To date, however, there are contradicting results regarding the effect that gangliosides lipids have on the aggregation of Aβ, with studies showing that gangliosides can both inhibit or accelerate Aβ aggregation. It has been suggested that the ganglioside lipid GM1, has been associated with promotion of Aβ fibrils.^28–30^ However, other studies have demonstrated that GM1 has the opposite effect and inhibits Aβ oligomerisation.^31,32^ Furthermore, experimental studies which incorporate computational and simulation results corroborate that ganglioside lipids can inhibit Aβ aggregation.^32^ However, it is important to note that not only have the underlying mechanistic processes not been fully investigated, but contradictory conclusions arising from different studies have confounded this issue further.^33^

Here, in order to address this and shed light on the interaction between ganglioside lipids and Aβ, we probe the aggregation kinetics of both Aβ42 and Aβ40 and find that gangliosides can delay the aggregation of Aβ42, while completely inhibiting the self-assembly of Aβ40. Through kinetic analysis we derive the microscopic rate constants behind these aggregation phenomena, and show that the rate of primary nucleation is greatly affected by the presence of gangliosides, being reduced by 3 orders of magnitude in the case of Aβ42. In comparison, we show that addition of lipids such as POPS, do not affect the primary nucleation rate of Aβ42 to the same degree as gangliosides. Furthermore, we perform viability assays of neuroblastoma cells and show that fibrils formed in the presence of gangliosides are less cytotoxic than fibrils formed in the absence of the lipid. Finally, we establish that gangliosides are able to form complexes with Aβ40/Aβ42, which was not the case for the other lipid tested (POPS), suggesting that such ganglioside-Aβ complexes may be key factors in the overall inhibition of amyloid-β aggregation. These results demonstrate the inhibitory and protective role that gangliosides can play in the aggregation of Aβ. More importantly, our results pave the way for new insights into the molecular mechanisms behind dementia and AD, and highlight how certain lipids can potentially be used for future healthcare applications.

## Results and discussion

### Ganglioside lipids inhibit amyloid-β aggregation

**Scheme 1:**
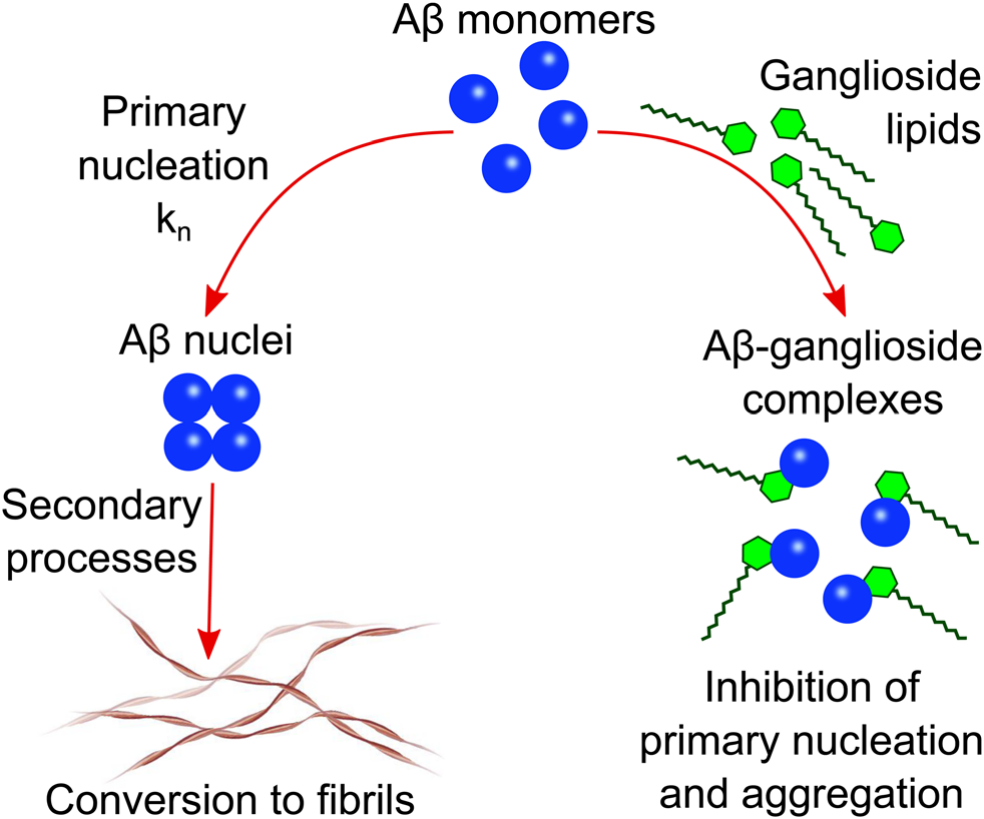
Schematic illustration of the proposed mechanism of Aβ inhibition in the presence of ganglioside lipids. Aβ forms a complex with gangliosides which in turn inhibits primary nucleation and subsequently the aggregation of Aβ into amyloid fibrils.

A general schematic illustrating how ganglioside lipids inhibit Aβ aggregation is shown in Scheme 1. The proposed mechanism which we describe involves an Aβ-ganglioside complex being formed which inhibits the aggregation of amyloid-β by decreasing the primary nucleation rate. The experiments leading to the findings supporting this proposed mechanism are described in the following sections.

**Fig. 1:**
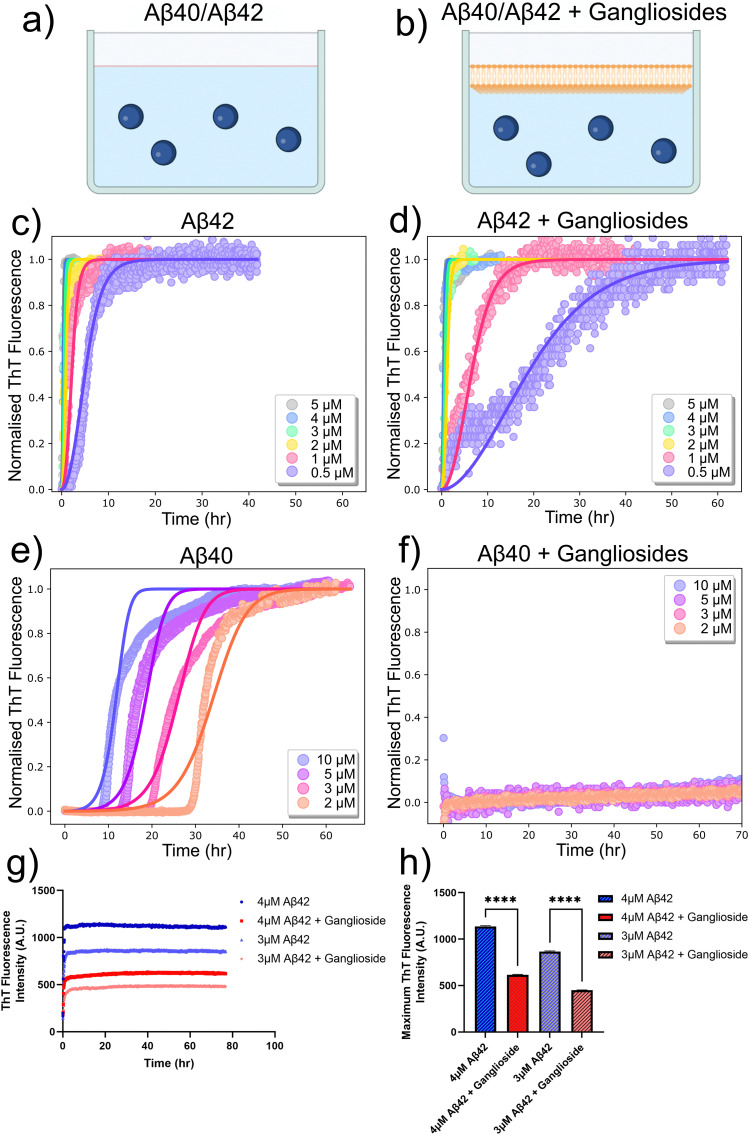
(a) and (b) Schematic representation of the experimental set-up used to probe the effect of ganglioside lipids on the aggregation of the Alzheimer's related peptide amyloid-β (Aβ). (c) and (d) Normalised ThT aggregation kinetics curves for various protein concentrations of Aβ42 in the absence and presence of gangliosides. Points correspond to experimental data while the solid lines correspond to the fits. (e) and (f) Normalised ThT aggregation kinetics curves for various protein concentrations of amyloid-β 40 (Aβ40) in the absence and presence of gangliosides. Total inhibition of Aβ40 aggregation was observed for samples containing a ganglioside interface. Points correspond to experimental data while the solid lines correspond to the fits. (g) Raw ThT aggregation kinetics for Aβ42 (3 and 4 μM solutions) in the absence and presence of gangliosides. (h) Maximum ThT fluorescence intensity values obtained from the corresponding curves in (g). Data are shown as mean ± SD. One-way ANOVA with Tukeys multiple comparisons test. ****p < 0.0001.

In order to investigate how interfaces and in particular gangliosides can affect amyloid-β (Aβ) aggregation, we varied the nature of the interface. This was done by either leaving the protein solution within the 96-well plate as is (Fig. 1(a)), or by adding a ganglioside lipid solution on top of each protein solution in the 96-well plate (Fig. 1(b)). This ensured that the ganglioside solution fully covered the protein phase and thus eliminated any other interfacial interaction. The kinetics of amyloid formation were then monitored by observing the increase in fluorescence of Thioflavin T (ThT). This fluorophore has the characteristic of increasing its quantum yield when bound to β-sheet rich structures, and therefore an increase in fluorescence intensity directly correlates with an increase in aggregated structures.

The resulting kinetic curves (which have a characteristic sigmoidal curve) show the build-up of fibril mass over time. In these experiments, a protein concentration range from (0.5 μM to 5 μM) was investigated. For all conditions, three repeats were conducted. It is clear from the results in Fig. 1(c) and (d), that in the absence of gangliosides, *i.e.* when amyloid-β 42 (Aβ42) is left to aggregate on its own, faster aggregation kinetics are observed (Fig. 1(c)) than when gangliosides are added to the top of the aggregation assay (Fig. 1(d)). This inhibitory effect can be easier seen for the lowest concentration of protein solutions used (0.5 μM). For this protein concentration, the half-time of aggregation increases from around 6 hours (Fig. 1(c)) to 30 hours (Fig. 1(d)) when ganglioside lipids are added to Aβ42. The kinetic data were normalized, using the formula: *y*_i_ = (*a*_i_ − min(*a*))/(max(*a*) − min(*a*)). This allowed the data to be normalized from 0 (lowest value) to 1 (largest value).

Moreover, we quantified these kinetic curves by employing the use of a chemical kinetics framework which allowed us to interpret the aggregation profiles in terms of the rate constants of the underlying microscopic steps of aggregation. Such steps include primary nucleation, fibril elongation, and secondary processes such as fragmentation and surface catalysed secondary nucleation. The rate constants which correspond to these microscopic steps are *k*_*n*_, *k*_+_, and *k*_2_ which are the primary nucleation rate, the elongation rate, and the secondary nucleation rate constant, respectively. *n*_c_ corresponds to the reaction order of the primary process and *n*_2_ is the reaction order of the secondary pathway. It is known that Aβ aggregates *via* secondary nucleation.^34,35^ Therefore, a secondary nucleation dominated model was used to fit the data for protein samples both in the absence and presence of the ganglioside interface. It was found that in the absence of gangliosides the elongation and secondary nucleation product, *k*_+_*k*_2_, obtained through this analysis was *k*_+_*k*_2_ = 1.20 × 10^19^ M^−3^ h^−2^, while the elongation and primary nucleation product was found to be *k*_+_*k*_*n*_ = 4.32 × 10^11^ M^−2^ h^−2^. Conversely, in the presence of a ganglioside interface, the corresponding combined rate constants were *k*_+_*k*_2_ = 1.23 × 10^17^ M^−3^ h^−2^ and *k*_+_*k*_*n*_ = 2.03 × 10^10^ M^−2^ h^−2^. It is clear from these results that the aggregation kinetics of Aβ42 proceed much faster in the absence of gangliosides.

Moreover, a similar assay was conducted for the smaller amyloid peptide, amyloid-β 40 (Aβ40). It was found that in the absence of gangliosides, the peptide aggregated *via* a secondary nucleation pathway, as expected,^35^ with rate constants (*k*_+_*k*_2_ = 5.86 × 10^14^ M^−3^ h^−2^ and *k*_+_*k*_*n*_ = 1.57 × 10^6^ M^−2^ h^−2^). The corresponding aggregation curves are shown in Fig. 1(e). However, upon addition of gangliosides, total inhibition of Aβ40 aggregation was observed across the same protein concentration range (2–10 μM), showing the potent ability of this lipid to delay and even stop the self-assembly of Aβ40. These results are shown in Fig. 1(f). Furthermore, the un-normalised aggregation curves of Aβ42 for a couple of protein concentrations with and without gangliosides were plotted (Fig. 1(g)). The ThT fluorescence intensity gives an indication of the amount of fibrils present in solution. For all protein concentrations tested, it was found that the solutions containing gangliosides always exhibited a decreased fluorescence signal, approximately by a factor of 2, suggesting the presence of fewer fibrils in these solutions. These data are summarised in Fig. 1(g) and (h).

### Surface-to-volume ratio affects amyloid-β aggregation by modulating the rate of heterogeneous primary nucleation

**Fig. 2:**
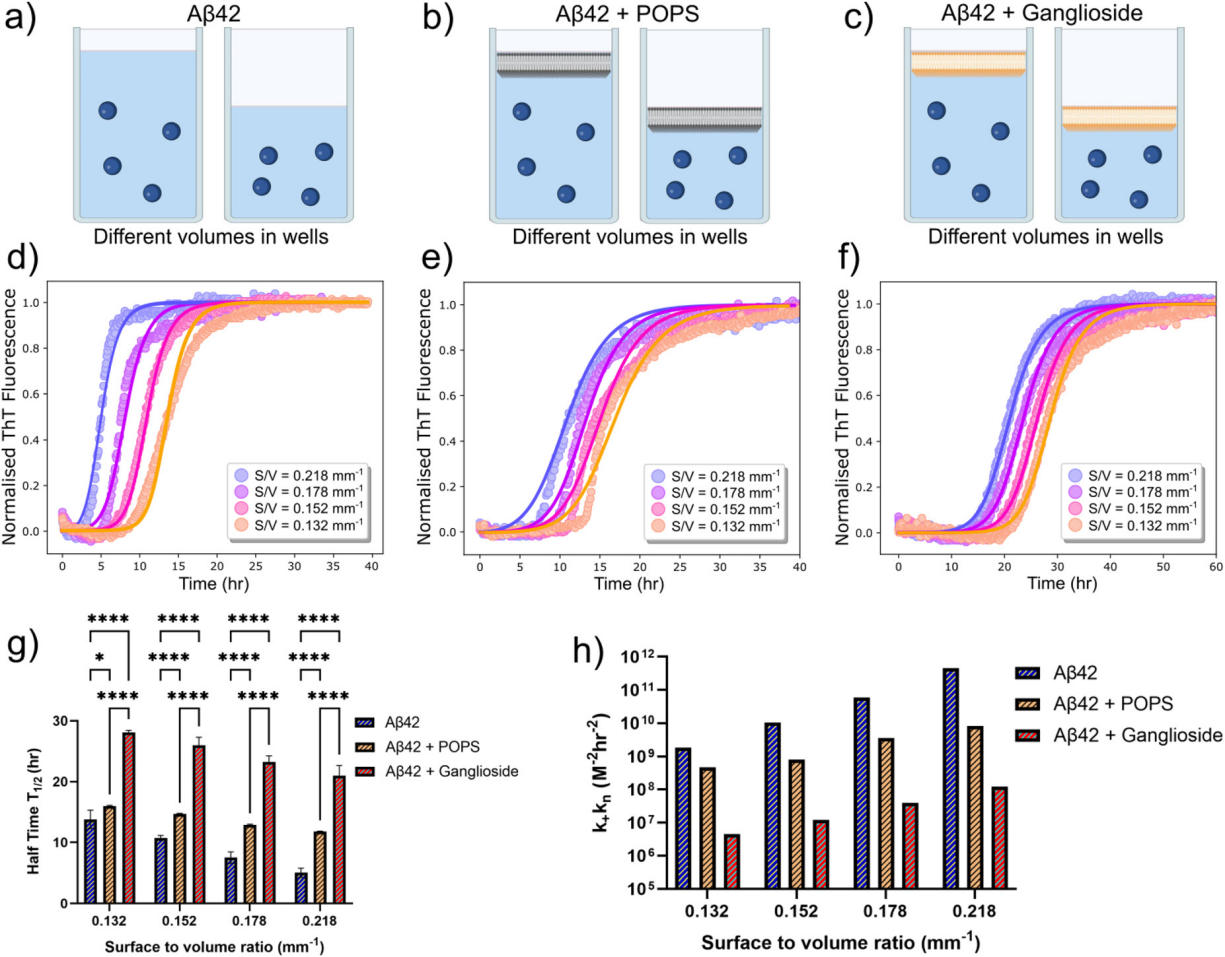
(a)–(c) Schematic representation of the surface-to-volume ratio (S/V) alterations to probe how surfaces affect heterogeneous primary nucleation of an Aβ42 solution (a) with the addition of a POPS (b) or a ganglioside (c) interface at the top of the protein solution. (d)–(f) Normalised ThT aggregation kinetics of a 0.5 μM Aβ42 solution alone (d) in the presence of POPS (e) and in the presence of gangliosides (f), for different S/V ratios. Points correspond to experimental data while the solid lines correspond to the fits. (g) Plot of half-time of the aggregation kinetics of a 0.5 μM solution of Aβ42 as a function of the S/V ratio for all three systems (alone, with POPS and with gangliosides). Data are shown as mean ± SD. Two-way ANOVA with Sidak's multiple comparisons test. ****p < 0.0001, *p = 0.0162 (h) plot of *k*_+_*k*_*n*_ as a function of the surface-to-volume ratio of a 0.5 μM solution of Aβ42 with and without the presence of the two lipids (POPS and gangliosides).

In order to determine the effect that surfaces can have on the rate of heterogeneous primary nucleation, we first investigated the effect of changing the surface-to-volume (S/V) ratio, as is shown in the schematic in Fig. 2(a). Kinetic experiments were thus conducted in systems with different volumes, where wells were filled with a range of 80–140 μL.^36^ This corresponded to a S/V ratio in the range of 0.218 to 0.132 mm^−1^. The results show that for the range of S/V ratios explored, a higher S/V ratio results in faster aggregation kinetics of Aβ42, Fig. 2(d). Since all parameters (other than the S/V ratio) remain constant within the solution, this effect can only be attributed to an increase in the primary nucleation rate. For all kinetic experiments three repeats were conducted. It should also be noted that due to the fact that a ganglioside interface completely inhibited Aβ40 aggregation (Fig. 1(f)), we chose to focus the S/V study primarily on the Aβ42 peptide.

We then proceeded to use the same experimental system (which included the variation in the S/V ratio) with the incorporation of lipids. In addition to investigating the effect of gangliosides on the aggregation kinetics, we also chose to include another lipid, 1-palmitoyl-2-oleoyl-*sn-glycero*-3-phospho-l-serine (POPS), as a control experiment. Phosphatidylserines (PS) are a class of phospholipids which are predominantly found at the cell membrane and form an integral part of it. PS is involved in a variety of cell signalling processes and provides structural stability for the cell membrane. For this reason, POPS was used as a control lipid. The lipids were added to the top of the protein solution (as shown in Fig. 2(b) and (c)) and the same volume range was explored. The kinetic graphs are shown in Fig. 2(e) and (f). The same trend was observed – higher S/V ratios result in faster aggregation kinetics. Moreover, while both lipids delayed Aβ42 aggregation, the inhibitory effect of gangliosides on the aggregation of Aβ42 was again observed and is clearly more prominent than that of POPS. Like before, each kinetic experiment was conducted three times. The half-time of each S/V ratio was plotted, the results of which are shown in Fig. 2(g). For all S/V rations, there is a decrease in the half-time and furthermore, for the same S/V ratio, addition of lipid delays the aggregation of Aβ42. In particular, for samples where gangliosides are present, the half-time of Aβ42 is massively increased, up to a factor of 2 to 3.

To quantify the effect that the S/V ratio has on the primary nucleation rate, we employed the use of chemical kinetics once again. The fitting was first performed for one S/V ratio, and all parameters other than *k*_+_*k*_*n*_ were kept constant thereafter. The *k*_+_*k*_2_ values used were taken from the analysis performed previously (Fig. 1(c) and (d)). The primary nucleation rate for each S/V ratio was then obtained and plotted in Fig. 2(f). This analysis (Fig. 2(f)) reveals that the elongation and secondary nucleation rate constants are independent of the S/V ratio and, more importantly, that the primary nucleation rate is greatly affected. In the absence of a lipid interface, the primary nucleation rate varies from 1.85 × 10^9^ M^−2^ h^−2^ to 4.51 × 10^11^ M^−2^ h^−2^. However, in the presence of a ganglioside interface, the primary nucleation rate varies from 4.43 × 10^6^ M^−2^ h^−2^ to 1.22 × 10^8^ M^−2^ h^−2^.

This tells us two things; that by changing the S/V ratio by less than a factor of 2, the primary nucleation rate constant is affected by more than 2 orders of magnitude, and for a given S/V ratio, the presence of a ganglioside interface can inhibit the primary nucleation rate by 3 orders of magnitude. Conversely, for a given S/V ratio, the presence of POPS only modulated the primary nucleation rate by 1 order of magnitude. These findings therefore suggests that different interfaces and lipids can strongly modulate the aggregation of Aβ42 through controlling the rate of primary nucleation.

**Fig. 3:**
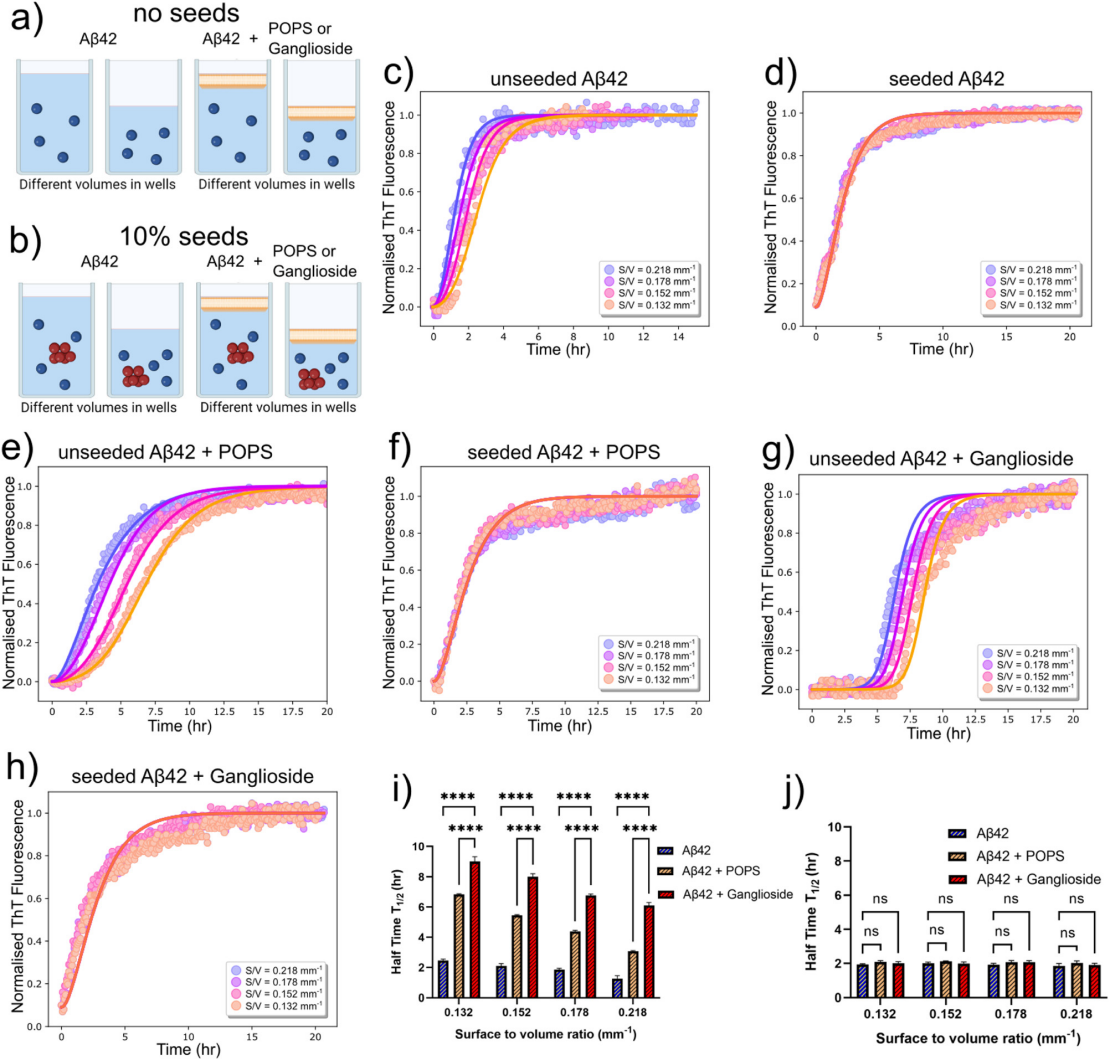
(a) Schematic showing the variation in the surface-to-volume ratio (S/V) with and without a POPS/ganglioside interface. (b) Schematic showing the same experimental setup as in (a), with the addition of preformed seeds. (c) Normalised ThT aggregation kinetics of a 1 μM Aβ42 solution in the absence of gangliosides, for different S/V ratios. (d) Normalised ThT aggregation kinetics with the same conditions as that in (c), with the addition of 10% seeds. (e) Normalised ThT aggregation kinetics of a 1 μM Aβ42 solution in the presence of POPS, for different S/V ratios. (f) Normalised ThT aggregation kinetics with the same conditions as that in (e), with the addition of 10% seeds. (g) Normalised ThT aggregation kinetics of a 1 μM Aβ42 solution in the presence of gangliosides, for different S/V ratios. (h) Normalised ThT aggregation kinetics with the same conditions as that in (g), with the addition of 10% seeds. In all cases, points correspond to experimental data while the solid lines correspond to the fits. (i) Plot of half-time of the unseeded aggregation kinetics in (c), (e) and (g) as a function the S/V ratio. (j) Plot of half-time of the seeded aggregation kinetics in (d), (f) and (h) as a function the S/V ratio. Data in both (i) and (j) are shown as mean ± SD. Two-way ANOVA with Sidak's multiple comparisons test. ****p < 0.0001, ns = non-significant.

We next sought to verify the conclusions derived from the kinetic analysis which suggested that interfaces modulate primary nucleation. This was tested by bypassing the primary nucleation step in the aggregation pathway, through addition of preformed seeds to the protein solutions in the 96-well plates (schematic shown in Fig. 3(a) and (b)). Assuming that the formation of nuclei predominately occurs at the interface, addition of seeds to the solutions would effectively negate any surface dependence on overall fibrillar growth, as the nuclei required to initiate the reaction would already be in solution. This implies that there should be minimal to no differences in kinetic behaviour when changing the S/V ratio. We tested this hypothesis for an Aβ42 solution with and without the presence of either POPS or gangliosides. In all cases, our prediction that the seeds would circumvent primary nucleation was verified and it is clear from the aggregation kinetic curves in Fig. 3(d), (f) and (h) that independently of the S/V ratio, similar kinetics are observed upon addition of 10% seeds. This is in stark contrast with the behaviour observed for the aggregation kinetics in the absence of seeds, Fig. 3(c), (e) and (g). Like before, three repeats were conducted for each kinetic experiment. Moreover, the half-time of each S/V ratio for unseeded Aβ42 aggregated in the absence and presence of either POPS or gangliosides is shown, where it is again clear that an increase in the S/V ratio enhances the kinetic process whereas addition of both lipids, but in particular ganglioside increases the half-time and therefore inhibits the overall reaction (Fig. 3(i)). Conversely, the half-time plot of the corresponding seeded solutions is shown in Fig. 3(j), where it is clear that for all cases the half-time has converged to the same value.

It should also be mentioned that due to the amphiphilic nature of lipids, there is a possibility that the lipid can partition into the protein phase. Due to gangliosides lipids having a much larger hydrophilic head compared to POPS, we anticipate that they would be more prone to partitioning into the protein solution. Furthermore, as we observe a clear surface-to-volume ratio effect (Fig. 2), it can be assumed that the surface is predominantly responsible for controlling protein aggregation, and any lipid partitioning into the aqueous solution does not significantly affected the protein aggregation kinetics. Additionally, this is further corroborated and reinforced by the seeded experiment, where independent of lipid choice, and surface-to-volume ratio, all the reactions occur at the same rate (Fig. 3).

### Morphological changes due to the presence of gangliosides

**Fig. 4:**
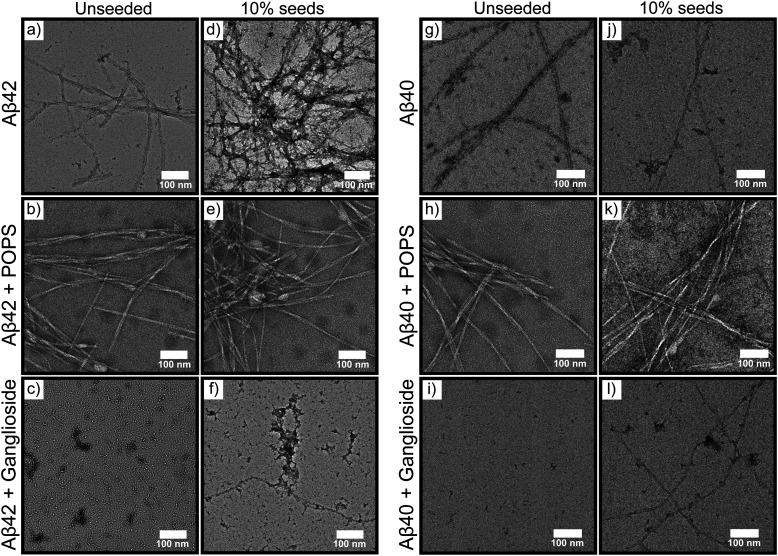
(a)–(c) TEM micrographs of 1 μM Aβ42 and (g)–(i) 1 μM Aβ40 following incubation of the peptides under different conditions. Alone (a) and (g), with POPS (b) and (h), and in the presence of gangliosides (c) and (i). (d)–(f) and (j)–(l) TEM micrographs of the corresponding systems with the addition of 10% pre-formed seeds.

As our results suggest that nucleation predominantly initiates at interfaces and that gangliosides have the ability to inhibit the aggregation of amyloid-β peptides, it stands to reason that the nature of the interface can modulate the morphology of the structures formed. In other words, samples with and without gangliosides could exhibit structural and morphological differences from each other. In order to explore this effect, transmission electron microscopy (TEM) was conducted on samples nucleated in the absence and presence of gangliosides and POPS. For all conditions tested, a 1 μM concentration of Aβ42/Aβ40 was used. The TEM results revealed that mature fibrils were formed for both Aβ42 (Fig. 4(a)) and Aβ40 (Fig. 4(g)) when aggregated in the absence of any lipid. Additionally, Aβ42 and Aβ40 fibrils were also observed for the samples co-incubated with POPS (Fig. 4(b) and (h) respectively). However, in the samples where gangliosides were present, mostly oligomers and protofibrils were seen for Aβ42 (Fig. 4(c)), and no ordered structures were observed for Aβ40 (Fig. 4(i)).

It should be noted that while POPS slightly inhibits the aggregation of Aβ42 (Fig. 2), the protein still aggregates and forms fibrils both in the absence and presence of POPS. The fact that POPS partially inhibits the aggregation process does not imply that amyloid fibrils do not form. Only in the case of ganglioside lipid with Aβ40, do we observe complete inhibition, which is why no aggregates were found in those samples (Fig. 4(i)). We do observe multiple fibrils for both Aβ42 and Aβ42 + POPS (Fig. 4(a) and (b) respectively), but not for Aβ42 + ganglioside (Fig. 4(c)). This therefore suggests that POPS does not reduce the overall number of aggregates. Moreover, it is also worth noting that the TEM images in Fig. 4 are indicative of each sample but do not represent a quantitative analysis and assessment of the fibrils. The images show that ganglioside lipids significantly reduce the number of Aβ aggregates formed, compared to when Aβ is left to aggregate on its own.

Moreover, TEM micrographs were acquired for both Aβ42 and Aβ40 samples co-incubated with preformed seeds. The TEM images show that under such conditions, gangliosides have a much smaller effect on the morphology of structures formed with mature fibrils being observed for all systems (Fig. 4(d)–(f) and (j)–(l)). This is due to gangliosides having the ability to modulate the primary nucleation of the aggregation process, which is circumvented if enough seeds are added to the solution. Additionally, it should be noted that we observed fewer fibrils in seeded samples where gangliosides were present, than in the corresponding samples which did not contain any gangliosides. Conversely, multiple fibrils were found in solutions which lacked any lipids and in samples were POPS was present. These observations are in agreement with our results for unseeded data, Fig. 1(g) and (h). Combined, these findings suggest that gangliosides not only inhibit Aβ aggregation, but also reduce the overall amount of fibrils present in the solution. Furthermore, circular dichroism (CD) spectra of Aβ42 by itself, Aβ42 with POPS and Aβ42 with gangliosides were taken. The data, shown in Fig. S1 (ESI†), suggest that Aβ42 alone (black curve) and in the presence of POPS (blue curve) form β-sheets. This is confirmed from the characteristic negative band at 217/218 nm. However, the sample where Aβ42 was aggregated in the presence of gangliosides (red curve) displayed two troughs, one at 217/218 nm and one at 207/208, suggesting that the species present in this sample are not pure aggregates, which confirms the TEM and ThT aggregation kinetic findings discussed above.

### Gangliosides inhibit amyloid-β aggregation within a cell mimicking lipid membrane mixture

**Fig. 5:**
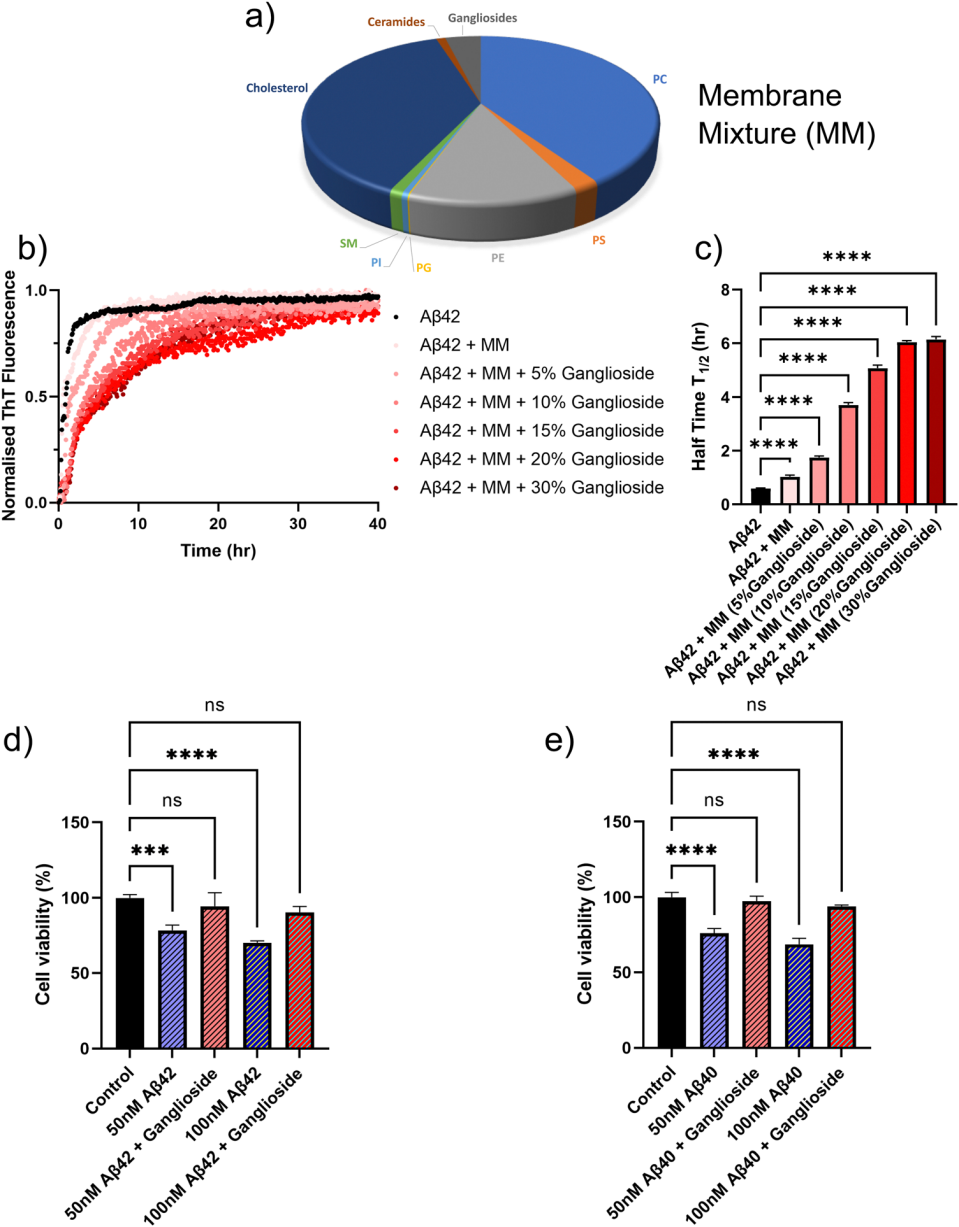
(a) Lipids used to mimic the membrane composition of a typical neuronal cell.^37^ (b) Normalised ThT aggregation kinetics of a 1 μM Aβ42 solution in the presence of a membrane lipid mixture (MM) with varying amounts of gangliosides. (c) Plot of half-time of the aggregation kinetics in (b) as a function of the amount of gangliosides present in the cell-mimicking lipid mixture. (d) and (e) Cytotoxicity of Aβ42 (d) and Aβ40 (e) of neuroblastoma cells (SH-SY5Y). Cell viability of SH-SY5Y cells was determined using an MTT assay following treatment with fibrils formed in the absence/presence of gangliosides. Data in both (d) and (e) are shown as mean ± SD. One-way ANOVA with Tukeys *post hoc* comparisons test. ****p < 0.0001, ***p < 0.001, ns = non-significant.

In order to further assess the inhibitory effect of ganglioside lipids on Aβ aggregation, we sought to mimic a cellular environment and monitor the aggregation kinetics. A mixture of different lipids, which corresponded to a typical membrane composition of a neuronal cell^37^ was prepared Fig. 5(a). A mixture of ceramides, cholesterol, sphingomyelin (SM), phosphatidylinositol (PI), phosphatidylglycerol (PG), phosphatidylethanolamine (PE), phosphatidylserine (PS) and phosphatidylcholine (PC) were used. We then sequentially doped the lipid mixture with varying amounts of ganglioside lipids, and monitored the aggregation kinetics for these different lipid-composition systems. It was found that in the absence of any lipid interface, Aβ42 aggregated the fastest (black points in Fig. 5(b)). However, upon addition of the lipid mixture which mimicked the cellular environment, we can already see a delay in the aggregation kinetics. Moreover, as the amount of gangliosides are increased within the lipid mixture, the aggregation of Aβ42 is further inhibited, in a dose dependent manner, with an inhibitory plateau being reached at around 20/30% addition of gangliosides (Fig. 5(b)). The half-time plots of the corresponding aggregation curves are shown in Fig. 5(c), where it is clear that the higher the amount of ganglioside within the lipid mixture, the larger the inhibitory effect. A similar trend was observed for the aggregation of Aβ40 (Fig. S2, ESI†) where in the absence of a lipid interface the peptide aggregated at a much faster rate compared to in the presence of a lipid mixture. However, upon dosing the lipid mixture with even a small amount of gangliosides (as low as 5%), total inhibition of Aβ40 aggregation was observed (Fig. S2, ESI†), which is in clear agreement with what was observed in Fig. 1(f).

### The cytotoxicity of SH-SY5Y neuroblastoma cells is reduced for amyloid-β fibrils aggregated in the presence of gangliosides

It is known that aggregates of Aβ possess cytotoxic properties, which have been clearly linked to neurodegeneration in Alzheimer's disease and other dementias. In order to obtain a more quantitative understanding as to how gangliosides may possibly protect cells against toxic amyloid fibrils, we performed assays investigating cellular viability. We assessed the cytotoxicity of fibrils formed both in the absence and presence of gangliosides with SH-SY5Y neuroblastoma cells (Fig. 5(d) and (e)). That is to say, Aβ was left to aggregate by itself, and in the presence of a ganglioside interface. The resulting aggregates were then added to the SH-SY5Y neuroblastoma cells. We then assessed the viability of the cells. It was determined that for both concentrations tested (50 and 100 nM), fibrils of Aβ42 formed in the absence of gangliosides were more cytotoxic that fibrils formed in the presence of ganglioside lipids. This can be seen Fig. 5(d). Moreover, in the case of Aβ40, 50 and 100 nM fibril samples were added to SH-SY5Y cells. Similarly to before, it was found that the samples where Aβ40 had been incubated in the presence of gangliosides there was minimal cytotoxicity, whereas the samples aggregated in the absence of gangliosides displayed reduced cellular viability in a dose dependent manner. These data are summarized in Fig. 5(e).

## Discussion

The results described in this paper provide the basis for understanding the inhibitory effect of ganglioside lipids on the aggregation of amyloid-β (Aβ). In the absence of a ganglioside interface, both Aβ42 and Aβ40 aggregate and self-assembly to form fibrillar structures, however, addition of gangliosides significantly inhibits the aggregation of Aβ42, while completely inhibiting the aggregation of the smaller amyloid fragment Aβ40 (Fig. 1). Moreover, by varying the surface-to-volume ratio (S/V) we show that primary nucleation occurs at the interface (Fig. 2(a) and (d)) and that addition of lipids at this interface not only inhibits the aggregation process (Fig. 2(e) and (f)), but more importantly, modulates and decreases the primary nucleation rate. Interestingly, the lipid POPS modulates the primary nucleation rate by only one order of magnitude whereas addition of ganglioside lipids decreases the primary nucleation rate by at least three orders of magnitude (Fig. 2(h)), which in turn inhibits the overall aggregation process.

Furthermore, transmission electron microscopy revealed that the morphologies of the fibrils is greatly affected by addition of gangliosides. Both Aβ42 and Aβ40 aggregated into mature fibrils in the absence of a lipid, and in the presence of POPS. However, in the presence of gangliosides, no aggregates were observed for Aβ40 (Fig. 4(i)), which confirms the kinetics finding in Fig. 1(d), while mainly protofibrillar structures were found for Aβ42 (Fig. 4(c)). Additionally, the cellular membrane environment of a neuronal cell was mimicked by combining a mixture of different lipids (Fig. 5(a)), and the aggregation kinetics of Aβ42/Aβ40 in the presence of this lipid mixture was monitored. It was found that not only does a lipid mixture delay the aggregation of Aβ, but doping such a lipid mixture with gangliosides further inhibited the self-assembly of the amyloid peptides (Fig. 5(b) and (c)), showcasing the importance that gangliosides present on neural membranes can play in the inhibition of Aβ aggregation. Finally, in order to assess the toxicity of aggregates formed in the absence/presence of gangliosides, cell viability assays were conducted with neuroblastoma (SH-SY5Y) cells. For both Aβ42 and Aβ40 it was observed that aggregates formed in the presence of gangliosides exhibited reduced toxicity to SH-SY5Y cells compared to fibrils formed in the absence of ganglioside lipids (Fig. 5(d) and (e)). This is in direct agreement with reports of the neuroprotective effects of gangliosides with cell cultures,^38^ rat models of Alzheimer's disease (AD)^39–41^ and even results from clinical trials.^39^

The fact that a lipid interface, and in particular gangliosides which are abundant in neuronal cells, can delay amyloid formation *via* the inhibition of primary nucleation, the fundamental first step in the aggregation process of Aβ, is of particular interest especially in light of the fact that ganglioside abundance may decrease with ageing.^22–24^ A schematic representation showing our overall findings is shown in Fig. 7. Moreover, given both the results obtained from our kinetic analysis and the cellular viability assays, what we can potentially conclude from our data is that gangliosides have a protective role against both the aggregation of Aβ peptides but also against cytotoxicity towards neuroblastoma cells (schematically represented in Fig. 7(d)).

A potential mechanism behind the inhibitory effect that gangliosides have on the aggregation of Aβ can be associated with the lipid's potential to form complexes with Aβ.^31,32^ It is known that particular gangliosides such as GM1, which is a component of the outer layer of the plasma membrane, specifically binds α-synuclein (a protein associated with Parkinson's disease) in order to promote an α-helix structure over β-sheet, the latter of which is the structure that aggregated α-synuclein adopts.^40,42,43^ It is therefore quite plausible that gangliosides can be implicated in binding and forming complexes with other proteins and peptides.

**Fig. 6:**
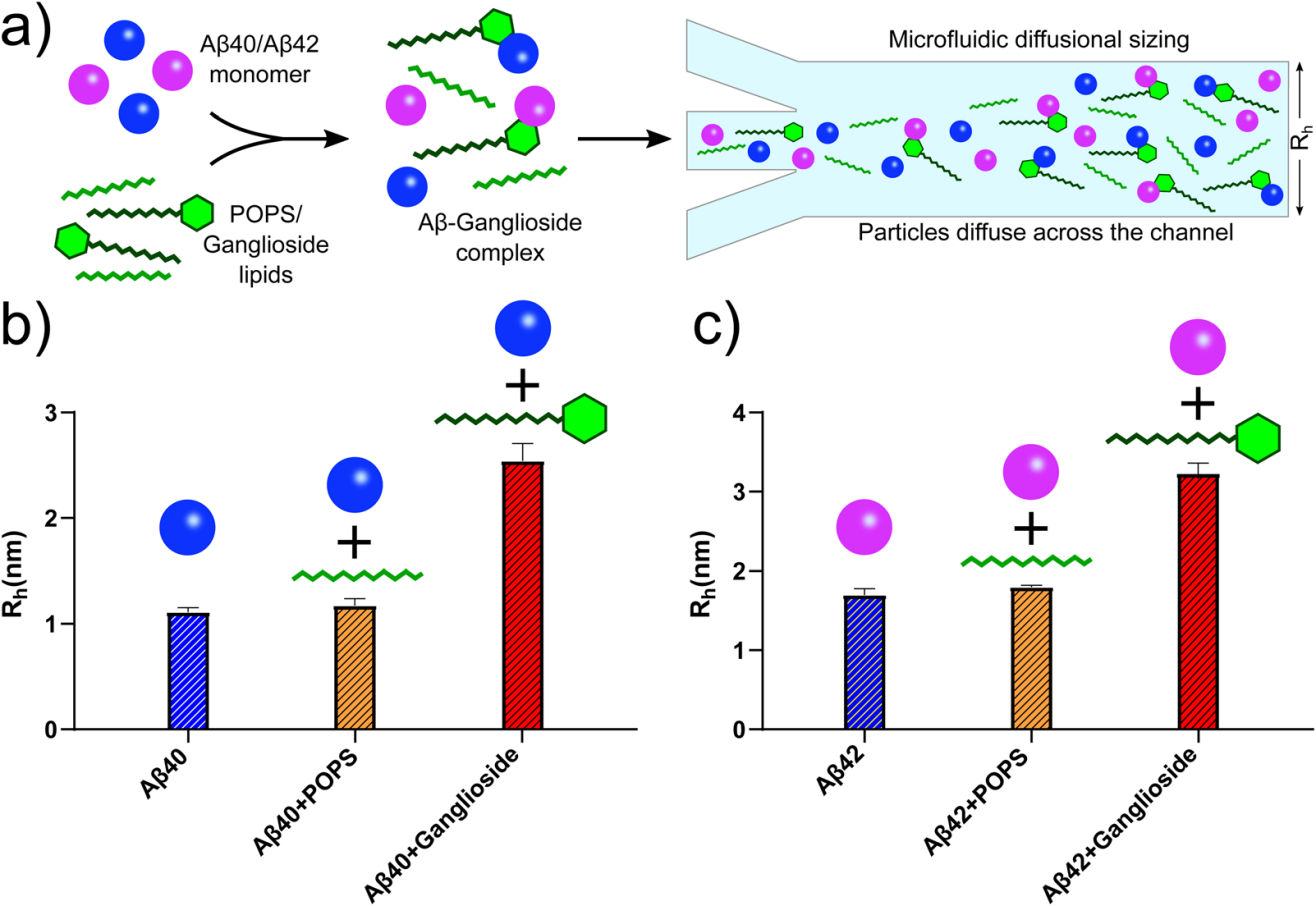
(a) Schematic representation of Aβ40/Aβ42 forming complexes with gangliosides but not with other lipids such as POPS. This was established using microfluidic diffusional sizing (MDS). (b) MDS results showing the hydrodynamic radii of Aβ40 monomers alone (blue bar), with POPS (yellow bar) and with ganglioside lipids (red bar). The change in hydrodynamic radius of Aβ40 with gangliosides but not with POPS suggests that the peptide formed a complex with gangliosides only. (c) Corresponding MDS results for Aβ42 using the same experimental protocol, monomer alone (blue bar), with POPS (yellow bar) and with ganglioside (red bar). The data again suggest that a complex between Aβ42 and ganglioside lipids was formed, but not between Aβ42 and POPS.

In order to investigate whether this is the case for Aβ, we conducted microfluidic diffusional sizing (MDS) on Aβ40/Aβ42 samples both in the absence and presence of lipids. MDS works by measuring the degree of molecular diffusion across a microfluidic channel, which in turn can be used to calculate the apparent hydrodynamic radius. The technique works by fluorescently labelling the molecule of interest. In this case, the Aβ peptides were labelled. The hydrodynamic radius of Aβ40/Aβ42 peptides was thus measured, Fig. 6(b) and (c) (blue bar). We then added POPS lipids to monomeric Aβ40/Aβ42 and the hydrodynamic radius of the Aβ peptides was measured. No detectable change was observed, implying that POPS does not bind with Aβ monomers (yellow bars in Fig. 6(b) and (c)). Finally, the same MDS experiment was conducted in the presence of gangliosides. An increase in the hydrodynamic radius was observed for both Aβ40 and Aβ42, which suggests that the ganglioside lipids bind to the Aβ peptide monomers and forms Aβ-ganglioside complexes. This is schematically represented in Fig. 6(a). Furthermore, studies have shown and hypothesised that GM1 gangliosides are in fact membrane binding sites for Aβ.^19,44^ Additionally, experimental^31^ and simulation results^32^ have recently corroborated that GM1 gangliosides form complexes with both Aβ40 and Aβ42, and by binding monomers this process can result in the inhibition of the aggregation pathway. In fact, the simulation results predicted that Aβ40 preferentially binds to GM1 gangliosides,^32^ which would explain why in our experimental results we observed complete inhibition of Aβ40 aggregation, while only partial inhibition of Aβ42. Therefore a potential mode of action is that ganglioside molecules create a complex with Aβ monomers and bind them in such a way that promotes a configuration whereby subsequent nucleation and fibrillar growth is impeded. The Aβ monomers are thus sequestered from the solution and the overall process of self-assembly is delayed. Such a mechanism could also be instrumental in how cells deal with extracellular Aβ. If gangliosides can form a complex structure with Aβ monomers, then cellular phagocytosis may be enhanced and Aβ can be cleared faster.^11^ If this mechanism is correct, this would also validate why ageing individuals, where ganglioside levels decline,^21–24^ are more likely to develop neurodegenerative diseases.

**Fig. 7:**
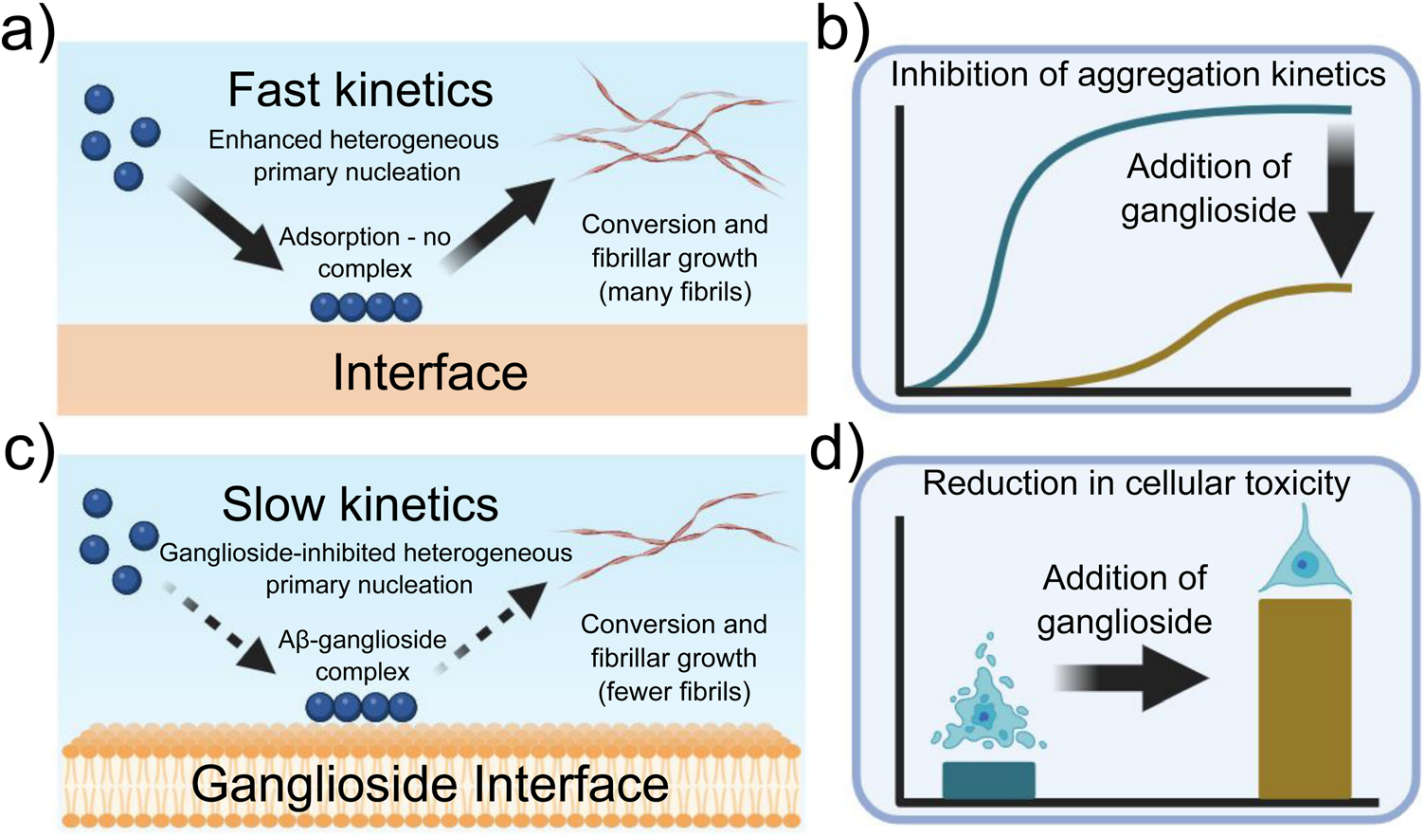
Schematic representation of the experimental findings described throughout this paper. (a) Primary nucleation and conversion of fibrils is enhanced in the presence of an interface, (c) while it is significantly inhibited when gangliosides are added, possibly due to the formation of an Aβ-ganglioside complex. (b) Aggregation kinetics of Aβ are inhibited in the presence of gangliosides, (d) while addition of lipids to the protein mixture before aggregation reduces cellular toxicity.

In conclusion, these findings further contribute to our understanding of how the aggregation of Aβ can be modulated by lipids which are predominantly found in the brain and how a reduction of certain lipids such as gangliosides can accelerate the primary nucleation rate and result in augmented formation of Aβ fibrils. Thus we shed light on the mechanistic processes behind some of the key initial steps underlying the aggregation process of Aβ42/Aβ40 in the presence of ganglioside lipids, that could be potentially used to inhibit the onset and proliferation of amyloid diseases such as Alzheimer's disease in the near future.

## Materials and methods

### Aggregation kinetics

In order to probe and monitor the aggregation kinetics of both Aβ42 and Aβ40 with and without the presence of a ganglioside interface, a previously established protocol was followed^36^ A 96-well plate (Corning 3881, Half-area) was used. This has a cylindrical shape and thus the cross-sectional surface area remains the same throughout the well, making it ideal to monitor how the surface-to-volume (S/V) ratio. Moreover, it is important to note that the plates used in all experiments are specifically coated in order to ensure that peptides/proteins do not adsorb to the walls of the wells. Thus interactions from the walls of the wells were not considered in this study.

In order to monitor the aggregation process, 50 μM Thioflavin T (ThT), a molecule that increases its fluoresce in the presence of β-sheets, was added to the solution. ThT fluorescence was monitored as a function of time using a microplate reader (FLUOstar-BMG labtech). The temperature during all experiments was 37 °C. All experiments were performed under quiescent conditions without agitation. Three repetitions were conducted for each kinetic run.

For the experiments involving a change in the S/V ratio, different volumes of peptides were pipetted into the wells (80, 100, 120, and 140 μL). This is schematically shown in Fig. 2(a)–(c).

### Amylofit kinetic analysis

All the kinetic data obtained was analysed using Amylofit.^45^ All data were initially normalised. The half-times were calculated from the time at which half the protein had aggregated, *i.e.* the time at which the normalised intensity reached 0.5. Each experiment was repeated three times and averaged before fitting. From the data, a secondary nucleation dominated model was found to be the best model to use, and thus this model was used throughout the study.

### Preparation of gangliosides, POPS and purification of Aβ42 and Aβ40

A concentration of either 1 mg mL^−1^ total ganglioside extract or 1 mg mL^−1^ POPS was used (Avanti Polar Lipids). In brief, ganglioside/POPS lipids were dissolved in chloroform and then left to evaporate overnight in a fume hood. Following this, 1 mL of hexadecane (Merck) was added to the dried film and vortexed for 5 min to ensure complete mixing. The lipid solution was then added on top of the protein solution in each well of the 96-well plate, as shown in Fig. 2(a)–(c). The aggregation kinetics of Aβ42 and Aβ40 were then monitored and investigated as described above. The method of purifying Aβ42 and Aβ40 was taken from a previously established protocol.^46^ Following purification, the peptides were lyophilised and then redissolved in 20 mM sodium phosphate buffer (pH 7.4 for Aβ42 and pH 8.0 Aβ40) at a stock concentration of 5 μM (Aβ42) and 10 μM (Aβ40).

### Seeded assay

For the experiments involving seeds, Aβ42 and Aβ40 were left to incubate (without the presence of a lipid interface) for one week at 37 °C. The aggregated peptides were then sonicated at 40% power for 30 s which resulted in the formation of seeds. A 10% seed solution was then prepared for both Aβ42/Aβ40 and experiments were performed accordingly as per the assays above. More information regarding the aggregation assay can be found in the aggregation kinetics section above. This is schematically shown in Fig. 3(a)–(c).

### Lipid mixtures for the cell mimicking system

For the experiments involving the cell mimicking system, lipid films consisting of a combination of different amounts of POPC, POPS, POPE, POPG, PI4P, sphingomyelin, cholesterol and ceramides (Avanti Polar Lipids) were evaporated in a glass vial. Specific ratios of each lipid, which mimicked the neuronal cell membrane as described in the lipidomics studies conducted by^37^ were used to form the final lipid solution. Following evaporation, a lipid film was formed, to which hexadecane (Merck) was added, and the experiments were conducted in a similar manner as described above. The lipid solution was added on top of the protein solution and the aggregation kinetics were monitored using a microplate reader (FLUOstar-BMG labtech).

### Transmission electron microscopy (TEM)

Transmission electron microscopy (TEM) was performed using a Thermo Scientific (FEI) Talos F200X G2 TEM operating at 200 kV. TEM images were acquired using a Ceta 16M CMOS camera. TEM grids (continuous carbon film on 300 mesh Cu) were glow discharged using a Quorum Technologies GloQube instrument at a current of 25 mA for 60 s. Samples were placed onto the grid for 30 s. Following this, the samples were blotted and then negatively stained using a 2% uranyl acetate solution for 45 s. Finally, samples were washed with deionised water.

### Cell culture of SH-SY5Y cells

SH-SY5Y cells (Merck), derived from the SK-N-SH neuroblastoma cell line, were cultured using Dulbecco's Modified Eagle Medium (DMEM, Thermofisher Scientific), 10% foetal bovine serum (FBS, Merck) and GlutaMAX Supplement (Thermofisher Scientific). Following confluence, cells were seeded at a density of 10^5^ cells per cm^2^ in a 96 well plate. For experiments involving cell viability, Aβ42/Aβ40 fibrils were extracted from the aggregation experiments, and the fibrils were incubated with the cells overnight. To prepare the aggregates added to the cells, Aβ42/Aβ40 was first incubated either by itself, or in the presence of a POPS or ganglioside interface (see schematic representation in Fig. 2(a)–(c)). The resulting aggregates were then diluted to the desired concentrations (50 or 100 nM) and added to the cells to incubate. All steps were performed in a sterile environment.

### Cytotoxicity and cell proliferation using MTT assay on SH-SY5Y cells

The viability of SH-SY5Y cells following incubation with fibrils formed in the absence/presence of gangliosides was determined using a standard MTT cell proliferation assay (Merck). In brief, 10 μL of MTT reagent ((3-[4,5-dimethylthiazol-2-yl]-2,5-diphenyltetrazolium bromide) labelling reagent Merck) was added to each well, followed by incubation for 4 hours. Subsequently, 100 μL of solubilisation solution was added and the well plate was further placed in the incubator overnight at 37 °C and 5% CO_2_. The resulting absorbance of the solubilised formazan crystals was measured using a plate reader at 595 nm (FLUOstar Omega microplate reader BMG Labtech).

### Microfluidic diffusional sizing (MDS)

Microfluidic diffusional sizing (MDS) experiments were conducted using a previously established protocol.^47^ In brief, microfluidic PDMS devices (the master of which was fabricated using a soft lithographic process^48–53^) were firstly coated with 0.01% Tween 20. Devices were then equilibrated for 1.5 minutes, before the experiment was run. Flow rates of 100–300 μL h^−1^ were used. The peptides were fluorescently labelled with Alexa488. To check for peptide–lipid complex formation, peptides were first flown through the device and the hydrodynamic radius of both Aβ42 and Aβ40 was determined. Following this, a lipid solution consisting of either ganglioside or POPS was prepared in deionised water. The peptides were mixed with the lipid solutions at a ratio of 1 : 1. These peptide/lipid solutions were then flown through the MDS device. A change in the hydrodynamic radius can only be attributed to the lipid interacting and forming a complex with the peptides. This is because the peptide is labelled with the fluorescent marker and therefore any change in the fluorescent readout must be due to the lipid forming a complex with the Aβ peptides.

### Circular dichroism (CD)

Far-UV CD spectra were taken between 195 and 250 nm. A Chirascan system (Applied Photophysics) was used to record the spectra. All measurements were conducted at 25 °C using a quartz cell cuvette with a 0.1 cm path length. 3 spectra were recorded for each sample, before being averaged. The protein concentration used for all samples was 1 μM.

## Author contributions

Z. T. conceived the study. Z. T. and A. K. J. conducted all experiments. Z. T. wrote the initial draft of the manuscript. All authors revised and contributed to the final version of the manuscript.

## Data availability

All original data is available from the corresponding authors upon reasonable request.

## Conflicts of interest

The authors declare no competing interests.

## Supplementary Material

CB-006-D4CB00189C-s001
