# Supplementary material for: Ganglioside lipids inhibit the aggregation of the Alzheimer's amyloid-β peptide
Source: RSC Chem Biol. 2025 Mar 13;6(5):809–22. doi: 10.1039/d4cb00189c (PMC11915136; doi:10.1039/d4cb00189c)
Supplement: CB-006-D4CB00189C-s001 [file CB-006-D4CB00189C-s001.pdf]

Supplementary Information (SI) for RSC Chemical Biology.
This journal is © The Royal Society of Chemistry 2025

Ganglioside Lipids Inhibit the Aggregation of the Alzheimer's Amyloid-β Peptide

Zenon Toprakcioglu,[†,‡] Akhila K. Jayaram,[†,¶,‡] and Tuomas P. J. Knowles[*,†,¶]

†*Yusuf Hamied Department of Chemistry, University of Cambridge, Lensfield Road, Cambridge CB2 1EW, UK*

‡*These authors contributed equally*

¶*Cavendish Laboratory, Department of Physics, University of Cambridge, J J Thomson Avenue, Cambridge CB3 0HE, UK*

E-mail: tpjk2@cam.ac.uk

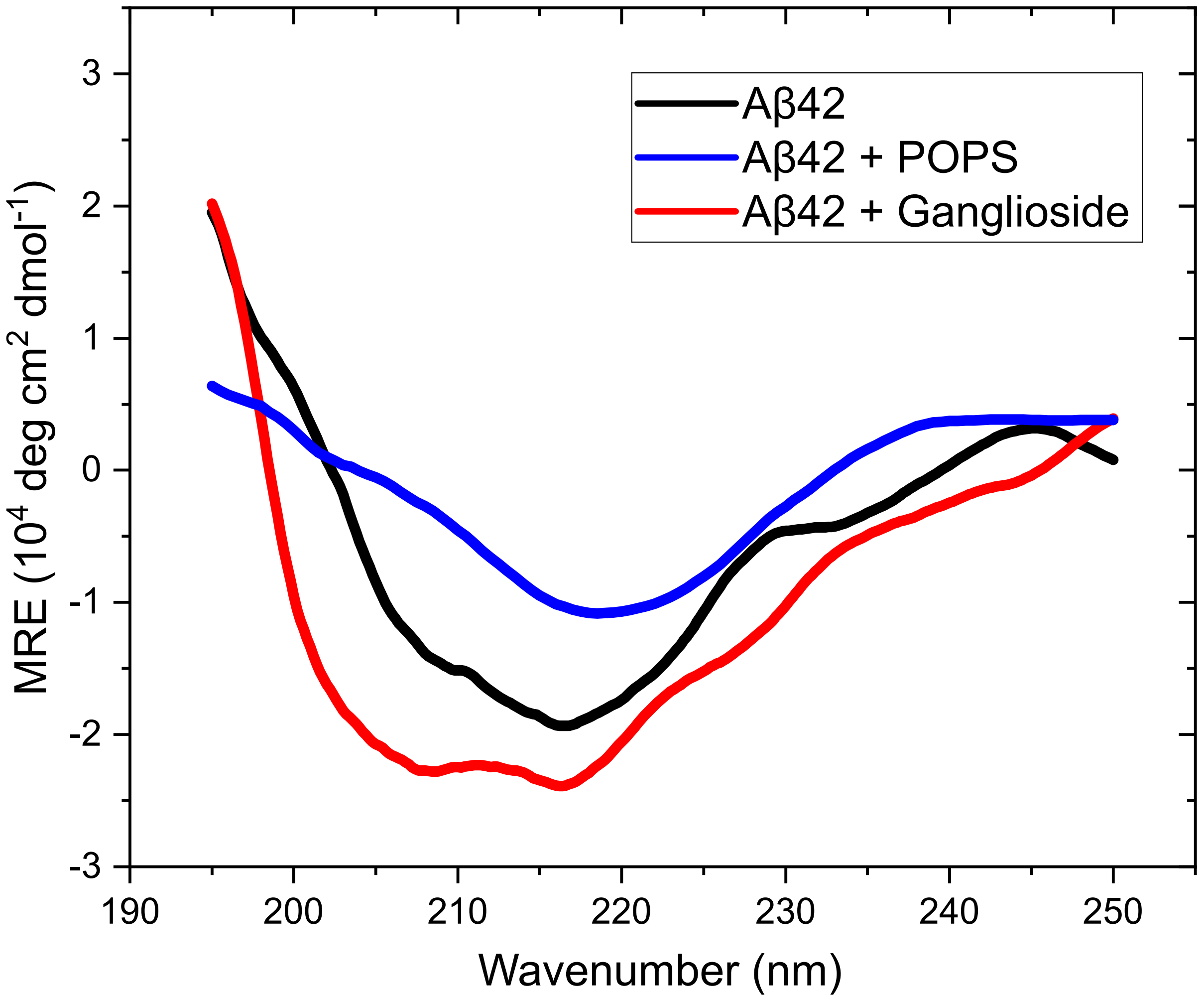

Figure 1: CD spectra of a 1 μM Aβ42 solution following aggregation in the absence of a lipid (black curve) with POPS (blue curve) and with gangliosides (red curve). The negative band at 217/218nm is characteristic of a β-sheet structure.

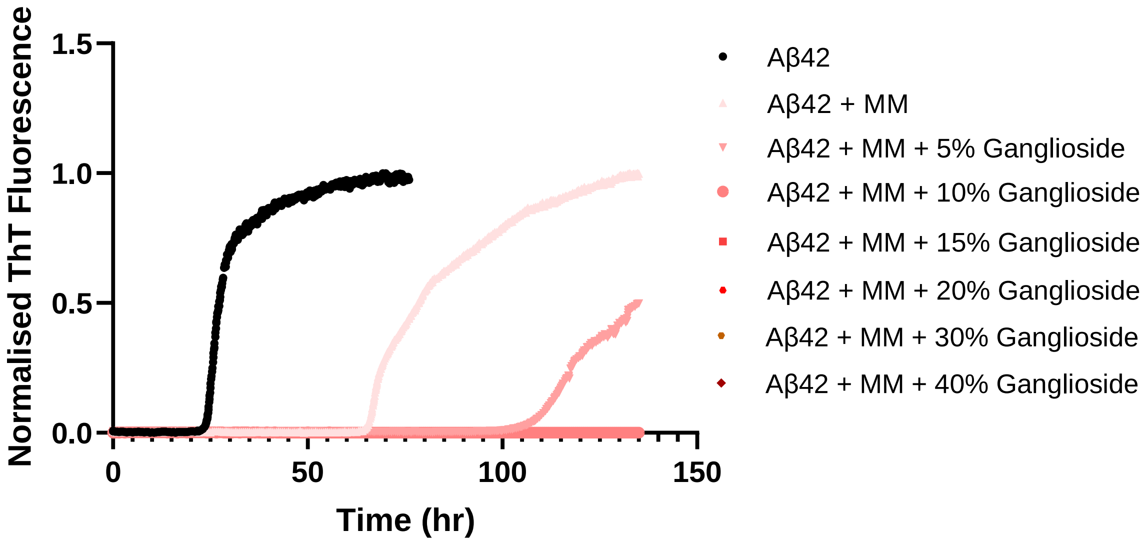

Figure 2: Normalised ThT aggregation kinetics of a 3 μM Aβ40 solution in the absence and presence of a lipid mixture with varying amounts of gangliosides.
